# Clinical chronobiology: a timely consideration in critical care medicine

**DOI:** 10.1186/s13054-018-2041-x

**Published:** 2018-05-11

**Authors:** Helen McKenna, Gijsbertus T. J. van der Horst, Irwin Reiss, Daniel Martin

**Affiliations:** 1 University College London Centre for Altitude Space and Extreme Environment Medicine, University College London Hospitals NIHR Biomedical Research Centre, Institute of Sport and Exercise Health, First Floor, 170 Tottenham Court Road, London, W1T 7HA UK; 2 Department of Molecular Genetics, Erasmus University Medical Center, Wytemaweg 80, 3015 CN Rotterdam, the Netherlands; 3 Division of Neonatology, Department of Paediatrics, Erasmus Medical Center, Rotterdam, the Netherlands; 4 Critical Care Unit, Royal Free Hospital, Pond Street, London, NW3 2QG UK

**Keywords:** Circadian rhythm, Chronobiology, Chronotherapy, Critical illness, Intensive care units

## Abstract

A fundamental aspect of human physiology is its cyclical nature over a 24-h period, a feature conserved across most life on Earth. Organisms compartmentalise processes with respect to time in order to promote survival, in a manner that mirrors the rotation of the planet and accompanying diurnal cycles of light and darkness. The influence of circadian rhythms can no longer be overlooked in clinical settings; this review provides intensivists with an up-to-date understanding of the burgeoning field of chronobiology, and suggests ways to incorporate these concepts into daily practice to improve patient outcomes. We outline the function of molecular clocks in remote tissues, which adjust cellular and global physiological function according to the time of day, and the potential clinical advantages to keeping in time with them. We highlight the consequences of “chronopathology”, when this harmony is lost, and the risk factors for this condition in critically ill patients. We introduce the concept of “chronofitness” as a new target in the treatment of critical illness: preserving the internal synchronisation of clocks in different tissues, as well as external synchronisation with the environment. We describe methods for monitoring circadian rhythms in a clinical setting, and how this technology may be used for identifying optimal time windows for interventions, or to alert the physician to a critical deterioration of circadian rhythmicity. We suggest a chronobiological approach to critical illness, involving multicomponent strategies to promote chronofitness (chronobundles), and further investment in the development of personalised, time-based treatment for critically ill patients.

## Background

Critical care medicine aims to maintain homeostasis in patients undergoing extreme physiological stress, for which they are no longer able to compensate. To this end, we strive to maintain physiological measures within parameters that we hope will promote optimal cell function and lead to survival; refining our targets as new evidence emerges. However, one fundamental facet of life has failed to form part of our concept of a patient’s well-being: the innate rhythmicity of their biological functions. We are all instinctively aware of the diurnal nature of our own behaviour throughout a 24-h period. We recognise the physical and mental affliction associated with its disturbance (e.g. jet lag and night shift work), but we give little consideration to the impact of these rhythms on the patients we treat. Most biological mechanisms fluctuate according to a circadian rhythm. The conservation of circadian rhythms across species points towards the survival advantage they convey, including: preparing an organism for daily recurring, and thus predictable, demands, such as the cyclical environmental changes in light, temperature and food availability imposed by the day–night cycle; and temporally segregating conflicting processes, such as feeding and physical activity, or sleeping and waking. The study of these cyclical phenomena, known as chronobiology, is growing in prominence throughout diverse disciplines, as their pervasive nature becomes apparent. During the extreme stress of critical illness, patients are particularly vulnerable from further impairment of cell function resulting from circadian rhythm disturbance. Critical care traditionally focuses on the “normalisation” of physiological indices, despite a limited evidence base [1, 2], but preservation of circadian physiology is not part of clinical practice. It is possible that neglecting the influence of circadian rhythmicity could contribute to the apparent lack of benefit from the majority of critical care targets tested in randomised controlled trials. In this review we introduce the basics of clinical chronobiology and propose the potential value to be gained from clinicians applying these new concepts to their daily practice. We propose that promotion of healthy circadian rhythms may constitute a future target for critical care medicine.

## The molecular networking of circadian rhythms

Our planet rotates through 360° every 24 h, creating an inescapable oscillation between light and darkness. Most life on Earth evolved under the influence of this day–night cycle, leading to the parallel oscillation of behaviour, physiology and metabolism throughout the 24-h period (diurnal rhythms). These rhythms are generated by cell-intrinsic molecular clocks with a periodicity of approximately (circa) 24 h (diem), for which reason they are called “circadian” clocks. Temporal compartmentalisation of functions according to predictable daily fluctuations in the environment has been vital to the survival of even the most primitive organisms, such as the single-celled *Synechococcus cyanobacterium*, which separates its two fundamental metabolic processes (photosynthesis and nitrogen fixation) by time rather than place [3]. In complex organisms such as humans, every cell has its own circadian clock. The cellular circadian rhythm is generated via a molecular network of transcriptional–translational feedback loops, with one cycle taking approximately 24 h to complete [4]. In short, the *Circadian Locomotor Output Cycles Kaput* (*CLOCK*) and *Brain And Muscle ARNT-Like 1* (*BMAL1*) genes encode a heterodimeric transcription factor known as CLOCK/BMAL1 that activates E-box promoter containing genes, including the *Period* (*PER1* and *PER2*) and *Cryptochrome* (*CRY1* and *CRY2*) core clock genes and a variety of clock-controlled genes (CCG) that couple the circadian oscillator to physiological and metabolic pathways. Once formed, PER/CRY protein complexes translocate to the nucleus where they inhibit CLOCK/BMAL1-mediated transcription of E-box genes, including their own [5]. Between 5 and 20% of all gene transcription in mammalian cells is controlled by this molecular oscillator depending on tissue type [6]. The oscillating transcriptome directly influences most biological pathways, ultimately shaping measurable indices such as body temperature, brain wave activity, cardiovascular and respiratory functions, coagulation and immunity [7].

## Keeping time

Coordination of mammalian circadian timekeeping involves an integrated system composed of a central clock in the suprachiasmatic nucleus (SCN) of the hypothalamus, and peripheral clocks in virtually all other cells and tissues. The coordinated integration of central and peripheral molecular clocks ensures that functions in different tissues occur at an appropriate time of day, such as sleeping at night and metabolising ingested nutrients during the day in the case of diurnal animals. Without these molecular clocks, there would be no temporal framework for daily processes. Although circadian clocks continue to tick indefinitely, they require careful adjusting to keep in time. The central clock in the SCN fulfils a key role in this, as it integrates input from external cues, known as Zeitgebers, to synchronise with the planetary light/dark cycle. Whereas light, perceived through the eye, is the strongest Zeitgeber for daily clock resetting (photoentrainment), other cues include temperature, feeding, exercise and social interaction. In turn, the SCN maintains synchrony of peripheral clocks through humoral and neural stimuli. Resetting the circadian clocks to alterations in Zeitgebers scheduling takes time [8] and sudden environmental changes result in cellular rhythms being temporarily out of step with demands imposed by the environment (external desynchronisation). This phase shift is experienced as jet lag when we rapidly cross longitudes and it takes approximately 1 day to adapt to each hour time zone crossed. Clocks in different tissues adapt to disturbance at different rates, leading to internal desynchrony, like the individual instruments of an orchestra playing independently [9]. The hormone melatonin appears to play a role in both external and internal synchronisation. Its secretion from the pineal gland is inhibited by bright light (detected by non-visual photosensitive retinal ganglion cells), hence circulating melatonin levels are very low during the day [10]. Melatonin was previously thought to regulate the sleep/wake cycle, but its secretion follows a similar pattern in nocturnally active animals (i.e. increasing melatonin levels at the beginning of the dark period) and may be more accurately considered the “hormone of the dark”.

### Chronotypes

Whilst most humans follow a pattern of daytime wakefulness and night-time sleeping, there is natural variation in preferred time to wake and sleep. Chronotype is the behavioural expression of the set point of an individual’s circadian rhythm. It describes their sleep–wake cycle in relation to time, and the two ends of the chronotype spectrum are called “morningness” and “eveningness”. Morningness is characterised by early-morning waking, with mental and physical performance peaking in the first half of the day, and going to sleep relatively early; eveningness is the opposite. Chronotype can be determined by questionnaires such as the Munich Chronotype Questionnaire and the Horne–Ostberg Morningness–Eveningness Score [11]. A comprehensive and well-controlled study demonstrated that questionnaires such as these closely correlate to dim-light melatonin onset time, probably the most reliable measure of central circadian timing in humans [12]. There is a strong genetic component underlying this phenotype [13] and ignoring its influence is known to be detrimental to performance [14]. The eveningness chronotype has been associated with a predisposition to diabetes, metabolic syndrome, high body mass index and sarcopenia, as well as depressive disorders [15–17]. Knowledge of a patient’s chronotype may be useful not just in the assessment of disease risk, but also in tailoring the timing of Zeitgebers such as light, food, exercise and sleep in order to preserve their natural circadian rhythm and optimise their biological function.

## Chronopathology

Circadian rhythm disruption can be genetic or acquired, and its impact extends far beyond the fleeting symptoms of jet lag (Table 1), reflecting the range of functions which follow a circadian rhythm. Chronic circadian rhythm disruption has been linked with diverse pathologies, from metabolic disorders, obesity, diabetes, cardiovascular disease and cancer [18–21]. Genetic disruption results from mutations of the clock genes responsible for generating the cellular circadian rhythm. Such mutations result in a spectrum of sleep pattern disorders [22]. Given that clock genes regulate cell proliferation, metabolism, DNA repair and apoptosis, it is of no surprise that polymorphisms in these genes (including *PER1* and *PER2*) have been associated with cancer in both mouse models and humans [23]. *CLOCK* gene mutations have been implicated in the development of obesity in humans and may play a key role in metabolic syndrome, type 2 diabetes and cardiovascular disease [24, 25]. Alterations within clock genes are also related to alcohol consumption [26] and recreational drug rewarding in animal models [27], indicating the far-reaching consequences of disruption to this fundamental regulatory system.

**Table 1:** Consequences of ‘wrong thing–wrong time’ leading to circadian desynchronisation in critically ill patients

Circadian misalignment	Clinical outcome
Sleep–wake inversion	Sleep loss [113]
	Delirium [114]
	Poor compliance with physiotherapy and rehabilitation [115]
Continuous feeding at night time	Glucose intolerance [64]
	Dyslipidaemia [72]
	Metabolic dysfunction [71]
Invasive interventions at night time	Impaired wound healing [101]

### Environmental clock disruption

Living out of phase with one’s body clock will also induce circadian dysrhythmia, and has also been shown to result in pathology [28]. Night shifts create a complete inversion of the sleep–wake cycle, analogous to travelling across 12 time zones. Night shift workers have been found to have higher rates of a number of behavioural and health-related morbidities [29] including sleep disorders, obesity [30], metabolic syndrome [31] and cancer [32, 33]. Sleep restriction also disrupts the circadian rhythm and has a profound effect on metabolic control in healthy volunteer subjects [34]. We are currently facing an epidemic of environmental circadian rhythm disturbance, as modern work and social schedules forces people to live contrary to their chronotype (social jet lag) [35].

## Circadian dysrhythmia in critically ill patients

**Fig. 1:**
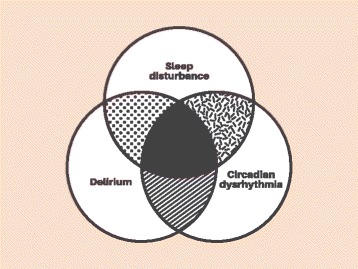
Venn diagram depicting inextricable relationship between circadian rhythm, sleep deprivation and delirium in critically ill patients

Critically ill patients are particularly susceptible to circadian disruption (dysrhythmia) due to mistiming, or total loss, of sensory cues disturbing the master regulation within the environment of the intensive care unit, and/or pathology affecting the peripheral clock mechanism at a cellular level. The latter phenomenon is imperfectly understood, but may relate to the inflammatory response, with septic patients seen to have abnormal patterns of melatonin secretion relative to non-septic ventilated patients [36]. The cellular effect outlasts the septic insult, with animal models of sepsis demonstrating rhythm disturbances for weeks after the insult [37], findings mirrored in human survivors of sepsis even after discharge from the intensive care unit. Rhythmicity can be affected in different ways, leading to a loss of amplitude, asynchronous timing or degradation to a completely chaotic pattern [38]. The clinical consequences of this aspect of critical illness are thought to be manifold but may have been neglected in the past due to the more obvious and immediately life-threatening features of physiological instability. The two most obvious manifestations of circadian dysrhythmia on the intensive care unit are the two complex and interrelated phenomena of sleep disturbance and delirium (Fig. 1). Sleep disruption is one of the commonest afflictions reported by survivors of critical illness [39]. It manifests as fragmentation and disruption of sleep architecture [40], and a displacement of the greater proportion of sleep to the daytime [41]. Disturbed sleep is not only a manifestation of circadian dysthymia, but also a driver of further disarray in peripheral clocks [42], creating a vicious circle of disruption. Of the many downstream effects of sleep disruption, its impairment of the immune system has the most obvious ramifications for recovery from critical illness [43]. Evidence is also emerging that ICU delirium, a syndrome independently associated with increased mortality and long-term morbidity from critical illness [44], and affecting up to 80% of critically ill patients [45], is a clinical manifestation of circadian dysrhythmia (Fig. 1). Delirium is associated with a reduction in peak amplitude of a urinary metabolite of melatonin (6-sulfatoxymelatonin) [46]. Circadian disruption and sleep disturbance often precedes delirium, this temporal link suggesting a possible causal relationship [47].

Many of the effects of critical illness circadian dysrhythmia may not be detectable clinically, but the diverse ramifications we see in chronic circadian dysrhythmia, outlined earlier, highlight the degree to which cellular function in different tissues is affected, beyond the overt symptoms of sleep disturbance and cognitive impairment. For example, mitochondrial oxidative phosphorylation itself exhibits circadian oscillation [48, 49], disruption of which may result in mismatching of cellular energetics to demand. In patients with sepsis where cellular bioenergetic failure is already threatened [50] and drugs such as propofol suppress mitochondrial respiration [51], such a discrepancy may be catastrophic for cell function. We propose that “chronofitness”, the correct alignment of peripheral and central clocks, should be a new target in the management of critical illness. The first step will be to identify and address the modifiable risk factors associated with the disruption of circadian rhythms in critically ill patients (Table 2), and implement strategies to prevent such disruption. Here we outline the rationale and evidence for a number of chronobiological strategies to improve outcome in critically ill patients.

**Table 2:** Suggested chronobiological approaches to preserve circadian rhythms on the intensive care unit (ICU)

Zeitgeber	Ideal ICU environment
Light	• Bright-light daylight hours (> 1000 lx) • Minimise light pollution at night (< 1 lx, consider) • Eye masks at night
Feeding	• Intermittent daytime feeding aligned to usual meal times • Avoid continuous and overnight feeding
Temperature	• Warmer environment during the day and cooler at night
Exercise rehabilitation	• Similar time each day • Take patient chronotype into consideration when planning schedule
Noise	• Minimise noise during the night time • Ear plugs at night
Medical and nursing interventions	• Minimise at night and cohort together • For non-sedated patients, allow patients to sleep according to their natural schedule, rather than a staff-based schedule
Sedative drugs	• Minimise use through regular review and avoid “sleeping tablets” • Consider melatonin (up to 5 mg) at 0900 h as circadian preserving sleep aide • When possible, give drugs at the time of day least disturbing to circadian rhythms

### The intensive care unit environment

Inside the intensive care unit, the usual schedule of Zeitgebers is obliterated. In the outside world, the variation between light and darkness ranges from 0.0001 lx on a moonless night to 0.25 lx during a full moon, 1000 lx on the most overcast day and 130,000 lx in brightest sunshine. At night, artificial light leaches into patient areas from many sources – room and corridor lights, the glare from monitors and torches used to measure pupil size (leading to mean night-time illumination of 10 lx in one study [52]) – whilst during the day indoor illumination rarely reaches that of the outside world even on an overcast day (mean daytime illumination 158 lx [52]). Excessive night-time light suppresses the release of melatonin, a key molecule orchestrating circadian rhythmicity in different tissues [53], and circadian disruption can be seen at a cellular level when cells are exposed to constant light [54]. Animal models of critical illness have demonstrated worse outcomes in the presence of circadian disturbance experimentally induced by constant exposure to light [55]. Imposing a light/dark cycle in a neonatal ICU accelerated body weight gain and shortened time to discharge in preterm infants [56]. “Chronofriendly” ICUs should aim to emulate normal day-time illumination levels with large windows, sufficient artificial lighting and perhaps incorporating blue-enhanced lighting in refurbishments and new builds [57]. A recent review summarised a number of randomised controlled trials in which morning bright-light therapy reduced delirium or improved sleep in acutely ill patients [58]. In a study published after this review, light therapy did not reduce the incidence of delirium in the critically ill and it is possible that it was less effective during early critical illness when patients were heavily sedated [59]. At night, providing sufficient light to perform critical tasks safely must be balanced against the negative effects of interruption of the patient’s light/dark cycle. Where possible, night light should be minimised or the patient’s eyes protected using eye masks. The use of lights and monitors that emit red rather than blue light at night may blunt inhibition of melatonin secretion [60]. Standards for morning light intensity during the day and night using luxmeters could be initiated.

Excessive night-time noise leads to sleep deprivation [61]. Noise reduction can be achieved by staff behaviour modification and the use of ear plugs in patients; the latter showing promising signs of being able to reduce ICU delirium [61]. Measurement of sound levels on ICU may help staff to understand which aspects of patient care create the most noise and facilitate the development of local guidelines to help minimise environmental noise pollution [62]. Other disturbances such as automated non-invasive blood pressure measurement, physical examination, turning and washing should be rationalised and cohorted at night, which as part of a bundle of interventions has been shown to improve sleep in the critically ill [63].

### Feeding

The consumption of food is probably the most powerful Zeitgeber for peripheral clocks. Most mammals do not eat at night and enter a period of natural fasting, during which time they switch from using primarily glucose as a fuel source to ketone bodies [64]. Yet it remains common practice to feed critically ill adults continuously, whether via the enteral or parenteral route. Time-restricted feeding is a well-described intervention that has a profound effect on the circadian rhythm and may be a powerful tool in the prevention of metabolic disorders [65, 66]. Much of the benefit seen in animal models exploring this line of work appears to relate to the maintenance or entrainment of the circadian rhythm [67]. Numerous animal model studies have demonstrated that changes to normal feeding schedules have significant metabolic consequences [68], including reduction in the amplitude and an alteration of phase in hepatic metabolite levels [69]. Daytime feeding in nocturnally active mice shifted the liver clock by 180° and resulted in significantly decreased survival rates in a sepsis model, in comparison to mice fed at night [70]. Eating out of phase with the SCN rhythm destroys the normal relationship between this central timekeeper and the peripheral clocks, and the resulting conflict manifests as gastrointestinal and metabolic disease [71]. This has been proposed as a mechanism for the increased incidence of obesity and metabolic disorders in shift workers [72]. A further interesting twist is the bi-directional circadian relationship between the gut microbiome and its host [73], with the circadian rhythm of one influencing the other. Whilst studies have been undertaken to look at outcome differences between bolus versus continuous feeding in subsets of critically ill patients [74, 75], they did not consider circadian factors in their design. A recent and eloquent personal perspective on this topic by Paul Marik [76] highlighted that providing continuous protein or amino acid supplementation limits skeletal muscle protein synthesis whilst intermittent feeding promotes it. His article concludes by stating: “Continuous tube feeding is unphysiological and likely harmful and should be abandoned” [76].

### Physical activity

Loss of daytime physical activity may be absolute in severe critical illness, as the result of sedation, paralysis, muscle weakness or critical illness polyneuropathy or myopathy. Posture on the ICU is predominantly supine, rather than upright, for much of the day. There is a chronobiological argument for incorporating early basic patient mobilisation, even passively, through physiotherapy, at the times relevant to that patient. Some have suggested that early mobilisation may contribute to circadian health, and early mobilisation has been suggested as being an essential component of any strategy to reduce delirium in the critically ill [77]. Individual chronotyping (through questioning relatives) may be especially valuable here; perhaps increases in compliance with valuable physiotherapy could be achieved in awake patients, by targeting their morningness or eveningness.

### Sedation

Sedatives can be a necessary evil in critical care medicine, but their side-effect profile may extend beyond our current awareness [78]. Administration of sedatives to the critically ill worsens sleep patterns and delirium, and drives circadian dysrhythmia [79, 80], with desynchronisation of the rhythmical secretion of melatonin demonstrated in sedated, mechanically ventilated patients [81]. Benzodiazepines appear to be the greatest offenders in this group [81, 82]. When possible, minimising the use of sedative drugs is likely to be beneficial for critically patients [78], and will require the optimisation of analgesia. In particular, it is advisable to avoid the knee-jerk reaction to treat sleep disturbance with sedative medications, which do not provide the active biological functions of sleep, and exacerbate sleep disturbance and delirium in the critically ill.

## Implementing an integrated “chronobundle” to entrain faltering circadian rhythms in the critically ill

**Fig. 2:**
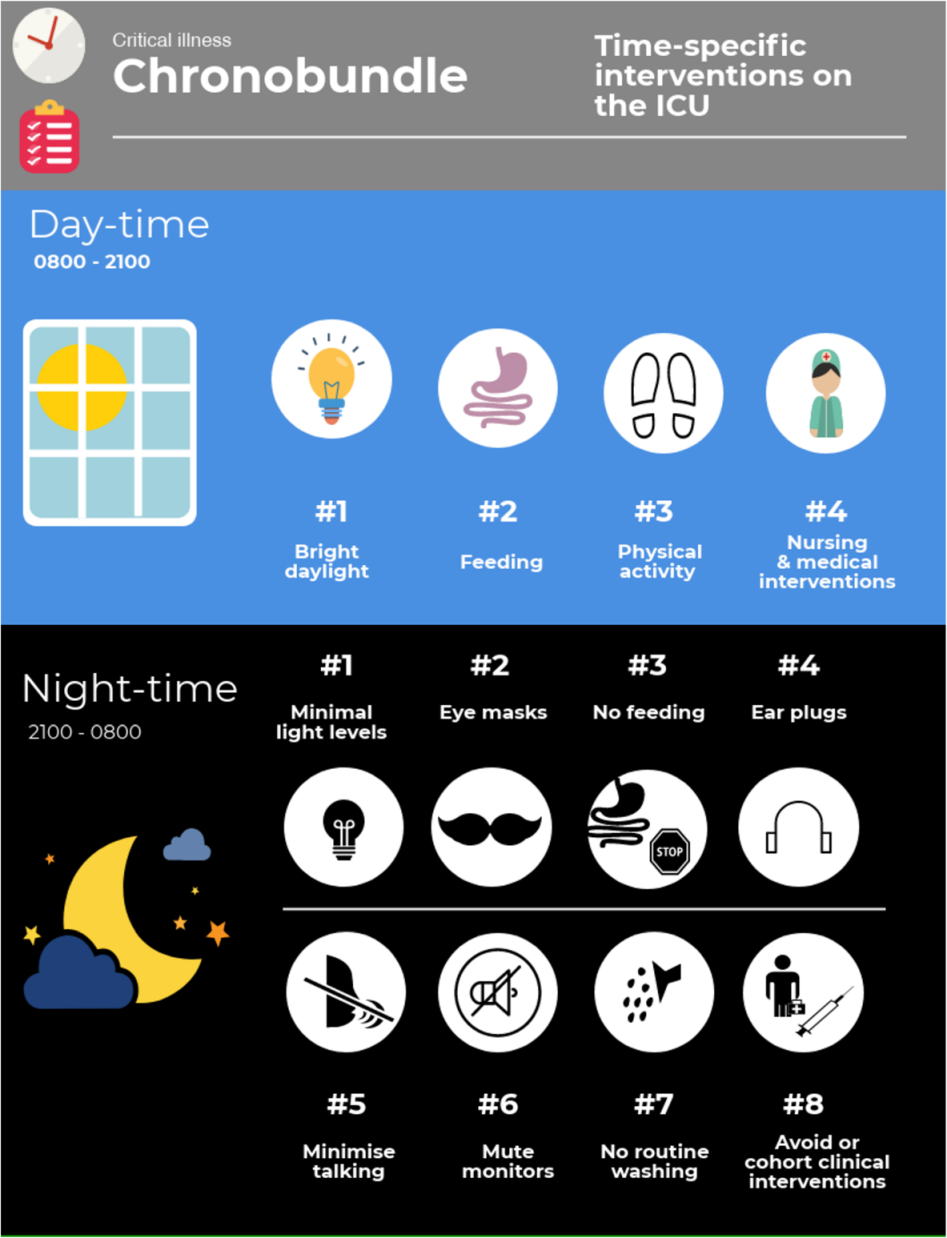
Schematic of a suggested “chronobundle” of care for critically ill patients. Attempt to maintain normal circadian pattern of activity even when patients are critically ill through simple multimodal scheduling and interventions. ICU intensive care unit

There is a biological rationale for keeping Zeitgeibers at their “expected” time to minimise harm from desynchronisation of peripheral and central clocks (Fig. 2). On the basis of the mechanisms and evidence from human and animal studies outlined, we propose a “chronobundle” to promote chronofitness as part of daily management of critically ill patients, summarised in Fig. 2. There is evidence that interventions may have greater benefit in combination, with a systematic review of multicomponent strategies based on environmental modifications demonstrating decreases in delirium incidence (odds ratio 0.47) [83]. Some chronobundle elements used to improve sleep in critically ill patients such as the use of ear plugs and eye masks have demonstrated benefit [84] and influenced circulating melatonin levels in a simulated critical care setting [85]. Individualising Zeitgeber timing according to a patient’s chronotype may further improve the impact of such interventions. This chronotype-specific approach has been explored as a performance enhancing tactic in both athletes [14] and college students [86]. Information regarding chronotype could be sought from close relatives and friends, and questionnaires exist to quantify responses from specific questions [11].

## Current challenges and future perspectives on the monitoring of circadian rhythms in the ICU

The volume of literature devoted to understanding the impact of circadian disruption on critically ill patients is small. In a setting in which heterogeneity of disease and treatments is sizable, linking cause with effect is challenging. Unless a patient’s circadian rhythm is accurately determined, it is impossible to determine how it may influence clinical outcomes. Currently, this is not possible outside the setting of complex research projects. Improving chronofitness in critically ill patients will require the ability to monitor circadian rhythms in the clinical setting. Measuring circadian rhythms, in real time and in different tissues, will be essential for identification of circadian dysrhythmias and for assessing the effect of interventions to improve clock alignment. As every physiological function has a circadian oscillation, each proffers a potential biomarker for tracking rhythmicity. In healthy volunteers and ambulatory patients, circadian rhythms can be mapped using activity monitors [87]. However, this has been shown to overestimate sleep time compared to polysomnography in the critically ill [88], presumably as actigraphy is unable to distinguish between true sleep and motionless wakefulness. It is usual to collect hourly physiological information from critically ill patients and the use of electronic data systems allows greater recording frequency of measurement variables providing a rich dataset of biorhythms. Although clinicians appreciate the importance of trends over time, we lack the terminology for describing or quantifying biological rhythms, or changes in their characteristics. Clinicians need to become more familiar with normal patterns of circadian oscillation in key physiological functions (e.g. the nadir of temperature, blood pressure and heart rate in the early hours of the morning), and to develop pattern recognition strategies to identify critical circadian disruption, similar to the manner in which we identify a rhythm disturbance in an electrocardiogram. A more sophisticated approach would require quantitative analysis of rhythms, perhaps via software able to process real-time physiological data, to identify: timing of peaks (or troughs) enabling phase shift determination; amplitude of variation, from peak to trough; and the consistency of the rhythm over days. Techniques to achieve this are commonly used in the experimental setting [89], but have yet to transfer across to clinical practice. Medical education in the future may need to provide clinicians with the mathematical competence to handle more complex data relating to biological rhythms, or to use algorithms for their interpretation. Attempts have been made to plot circadian rhythms using biomarkers including serum melatonin [90] and cortisol levels [91], urinary melatonin metabolites [36, 81], sleep–wake cycles using polysomnography [41] and hourly temperature and urine outputs [92]. A multitude of hormones related to metabolism levels are under circadian control such as insulin, ghrelin, leptin and growth hormone [93, 94]; these too could be used to help determine circadian profiles in the critically ill in a study setting [95]. Finding the optimum tool to monitor critically ill patients therefore remains a key to research progress in this area.

## Chronotherapeutics: delivering the right treatment at the right time

The ability to map an individual’s circadian rhythm conveys the potential ability to personalise critical care. For example, given the typical fluctuations of physiological variables throughout the 24-h period in health, we could evaluate the benefit of different physiological targets for day and night.

Monitoring circadian rhythms will be essential for the implementation of “chronotherapeutics”, the timing of an intervention or administration of a drug at the time of day where it is likely to have the optimum effect. Pharmacodynamics and pharmacokinetics show that predictable circadian oscillations manner [96, 97]. Whilst we tend to prescribe certain drugs at certain times of the day, it is generally without consideration of how each drug’s therapeutic profile may be affected by the time of day. Absorption, hepatic metabolism and renal excretion all follow a circadian rhythm [97], as do the biological processes on which drugs act. Chronotherapeutic strategies have proved beneficial in cancer treatment [98] where circadian-timed chemotherapy may improve outcomes. Optimising the timing of drugs with a narrow therapeutic index and significant circadian fluctuation, such as antibiotics, steroids and anticoagulants, could lead to significant clinical benefit from improvements in efficacy and minimisation of toxicity [97, 99]. Benzodiazepines phase-shift the clock according to the time of day at which they are administered [100], and thus the circadian disruption effect could be minimised by administering the drug at the correct time of day relative to the patient’s circadian phase.

Our defence from trauma and its accompanying stress response is also dependent on the time of day. Recently, it was reported that elective cardiac operations performed in the afternoon were associated with improved patient outcomes, which the authors related to changes in the oscillating gene expression of the nuclear receptor Rev-Erbα [101]. The time-of-day factor has also been implicated in the efficacy of wound healing in patients, with burn injuries incurred during the day healing up to 60% faster than those sustained at night, which may be explained by temporal patterns of fibroblast activity in a mouse model [102].

These preliminary data point towards the improvements in outcome that may be achieved by greater understanding of the temporal nature of healing responses, and highlight the need to develop a comprehensive temporal blueprint for different tissue rhythms to allow clinicians to plan interventions to take advantage of the predictable cycling of human immune and healing responses. Such a blueprint will have to take account of differing chronotypes.

### Pharmacological adjustment of circadian dysrhythmia

Melatonin is thought to align the phases of peripheral clocks in different tissues with that of the central SCN [103]. In humans, melatonin is released from the pineal gland, beginning from 2100 to 2300 h, peaking between 0100 and 0300 h, and reaching its nadir between 0700 and 0900 h (virtually a mirror image of cortisol release). Taken enterally, melatonin improves the quality of sleep [104], and there is some evidence for its effectiveness in treating jet lag [105]. A number of randomised controlled trials have investigated melatonin and melatonin receptor antagonists in critically ill patients [106]. They have shown initial success in improving sleep quality and reducing delirium in some subsets of critically ill patients [107–110]. However, no large-scale randomised controlled trials have been conducted in this area and this is clearly a priority area for future research.

In the future we may be able to target the molecular clockwork itself with pharmacological agents. Synthetic ligands are currently under investigation for Rev-Erb (α and β) and retinoic acid receptor-related orphan receptors [111]. They have a particular potential for the treatment of metabolic syndrome [112], but as this field advances the scope of synthetic ligands may widen to include more fields of medicine, including critical care.

## Conclusions

Circadian rhythms are currently low on the list of physiological priorities during ICU ward rounds. We have argued that recognition of the influence of this universally important system, and adoption of chronobiological strategies, has the potential to improve patient outcomes [38]. Circadian rhythmicity represents one of the oldest survival strategies, present in all life-forms on earth. Traditional treatment of critical illness ignores this fundamental physiological function, with many of our interventions inadvertently obliterating it. A new chronobiological approach to patient care would involve: education for clinical staff, regarding the recognition of circadian rhythms and dysrhythmias; development of technology to measure circadian rhythms quantitatively in critically ill patients; and investing in multicomponent strategies (chronobundles) to preserve or restore the synchronicity, phase and amplitude of circadian rhythms. For the latter, pharmacological solutions remain untested, but the non-pharmacological components of these bundles are simple and logical interventions of relatively low cost. Any chronotherapy will ultimately need to take account of time of day and individual patient chronotype.
